# Aggressive, Widely Metastatic Inflammatory Myofibroblastic Tumor in an Adult Presenting as an Obstructing Endobronchial Mass

**DOI:** 10.7759/cureus.107763

**Published:** 2026-04-26

**Authors:** Zehra Rahman, Tiffany Scotto, Alexander Den Boef, Julie Gaudin, Oday Elmanaseer

**Affiliations:** 1 Internal Medicine, University of Florida College of Medicine – Jacksonville, Jacksonville, USA; 2 Hematology and Medical Oncology, University of Florida College of Medicine – Jacksonville, Jacksonville, USA

**Keywords:** alk, alk-positive, anaplastic lymphoma kinase (alk) tyrosine kinase inhibitor, imt, inflammatory myofibroblastic tumor (imt), obstructing mesenteric mass, pulmonary mass

## Abstract

Inflammatory myofibroblastic tumor (IMT) is a rare mesenchymal neoplasm, most commonly occurring in children and young adults. It is typically locally aggressive and rarely metastasizes. Adult-onset IMTs with extensive metastasis are exceedingly uncommon, particularly with large pulmonary involvement, and can mimic sarcoma or sarcomatoid carcinoma on imaging and histology. Anaplastic lymphoma kinase (*ALK*) positivity aids diagnosis and provides a potential therapeutic target.

A 56-year-old male presented with progressively worsening dyspnea, nonproductive cough, and right-sided chest pain. Computed tomography revealed a 9-cm right hilar and upper lobe mass causing complete obstruction of the right upper lobe bronchus, with extensive mediastinal lymphadenopathy. Bronchoscopic biopsy demonstrated a malignant spindle cell neoplasm with sarcomatoid and myxoid features. Immunohistochemistry was positive for *ALK-1*, CD68, and vimentin, and negative for epithelial, smooth muscle, neural, and melanocytic markers (AE1/3, p40, TTF-1, CK7, CK20, CDX2, SMA, desmin, SOX10, S100, and calretinin). Staging imaging revealed a mesenteric mass adjacent to the ascending colon. Biopsy of the colonic lesion showed morphologically and immunohistochemically similar features, supporting a diagnosis of metastatic IMT confirmed by multidisciplinary pathology review. The patient’s clinical course was rapidly progressive, and he died two months after initial presentation.

Adult-onset *ALK*-positive IMT with extensive pulmonary involvement and multiorgan metastases is exceedingly rare. This case highlights the diagnostic challenges posed by sarcomatoid morphology and underscores the importance of integrating histopathology, immunohistochemistry, and clinical correlation, with *ALK* testing providing both diagnostic confirmation and a potential therapeutic target.

## Introduction

Inflammatory myofibroblastic tumor (IMT) is a rare intermediate-grade mesenchymal neoplasm that most commonly affects children and young adults and typically arises in the lungs, abdomen, or pelvis. Histologically, IMT is characterized by myofibroblastic and fibroblastic spindle cells and inflammatory infiltrate [1]. 

Although IMT was historically classified as an inflammatory pseudotumor, advances in molecular pathology have demonstrated that it represents a true neoplasm. A significant proportion of IMTs harbor rearrangements involving the anaplastic lymphoma kinase (*ALK*) gene, which not only supports the neoplastic nature of the disease but also has important therapeutic implications, as *ALK* inhibitors have demonstrated clinical efficacy in unresectable or metastatic disease [1].

Clinically, IMTs exhibit a spectrum of biologic behavior ranging from indolent, localized tumors amenable to surgical resection to rare aggressive cases characterized by local recurrence, invasion, or distant metastasis. Reported recurrence rates range from approximately 10% to 25%, while metastases occur in fewer than 5% of cases [2]. While the majority of cases occur in children and adolescents, IMT in adults is uncommon and may be associated with more aggressive clinical behavior.

We present a rare case of widely metastatic IMT in a 56-year-old male who initially presented with an obstructing endobronchial mass and rapid clinical deterioration. This case highlights an unusual and aggressive manifestation of a typically indolent tumor and underscores the importance of recognizing IMT in the differential diagnosis of spindle cell neoplasms in adults, as well as the potential role of targeted therapy in advanced disease.

## Case presentation

A 56-year-old male with no significant past medical history presented with progressively worsening dyspnea on exertion, nonproductive cough, and right-sided chest pain for 10 days before presentation. He also reported decreased appetite and unintentional weight loss, though he was unable to quantify the amount. The chest pain radiated down the right upper extremity and was associated with numbness and tingling. He denied fevers, chills, or hemoptysis. The patient worked in construction and reported significant occupational exposure to concrete dust. He denied tobacco use and illicit drug use and reported only occasional social alcohol consumption.

On presentation, vital signs were notable for a blood pressure of 116/71 mmHg, heart rate of 79 beats/min, respiratory rate of 16 breaths/min, oxygen saturation of 97% on room air, and temperature of 97°F (36.1°C). Physical examination was notable for bilateral wheezing, more pronounced on the right. No rashes or lymphadenopathy were appreciated. Laboratory studies were largely unremarkable aside from leukocytosis of 16.25 ×10⁹/L with neutrophilic predominance.

**Figure 1:**
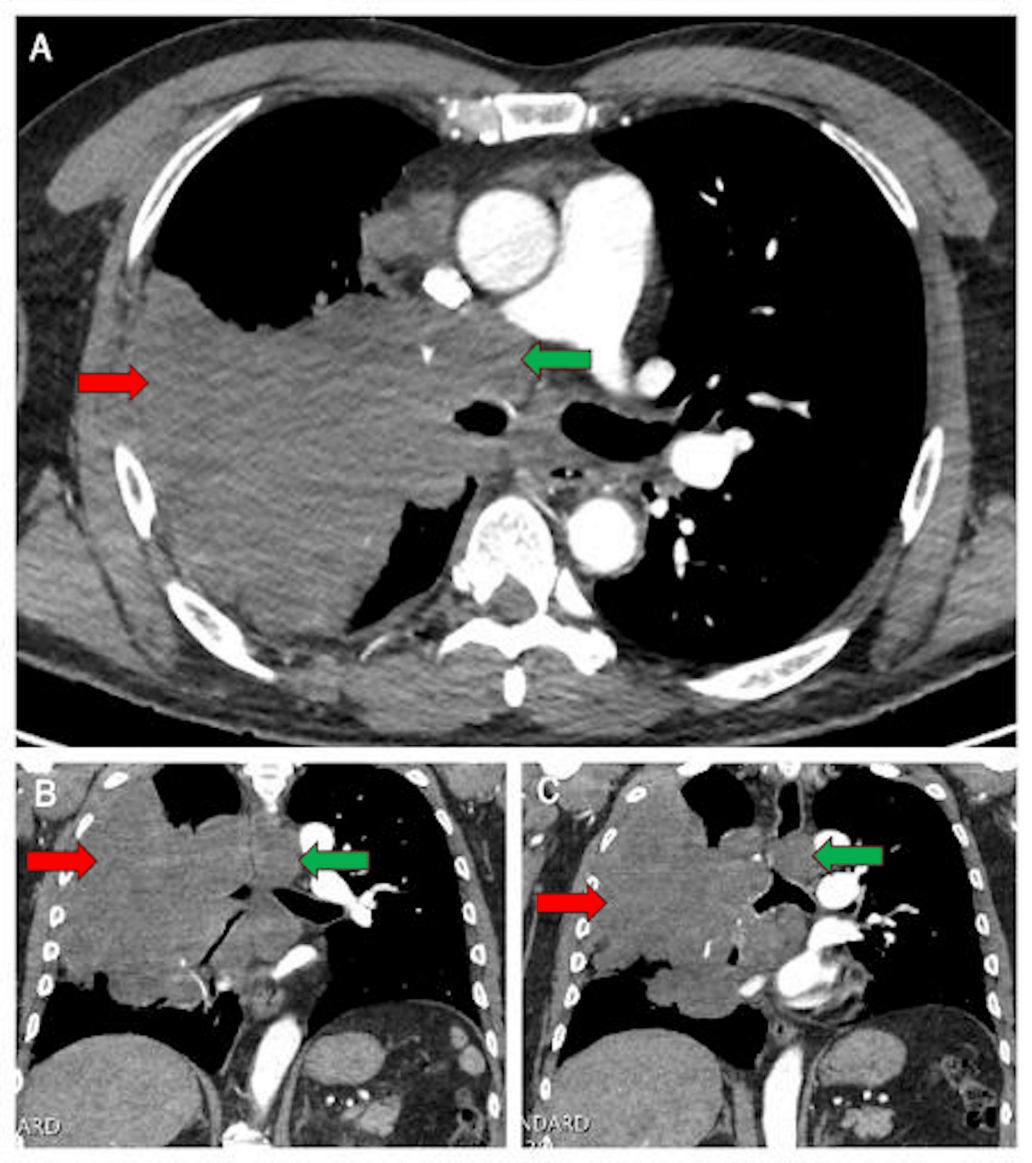
Contrast-enhanced computed tomography (CT) of the chest demonstrating the right hilar and upper lobe mass. Panel A shows an axial view, and panels B and C show coronal views. The mass is indicated with red arrows, and the associated mediastinal lymphadenopathy is highlighted with green arrows. The images demonstrate complete obstruction of the right upper lobe bronchus and extensive lymph node involvement.

Initial computed tomography (CT) of the chest demonstrated a segmental left lower lobe pulmonary embolus and a subsegmental left upper lobe pulmonary embolus. Imaging also revealed a 9-cm right hilar and upper lobe obstructing mass with extensive mediastinal lymphadenopathy involving multiple nodal stations. There was complete obstruction of the right upper lobe bronchus and severe narrowing of the bronchus intermedius (Figure 1).

**Figure 2:**
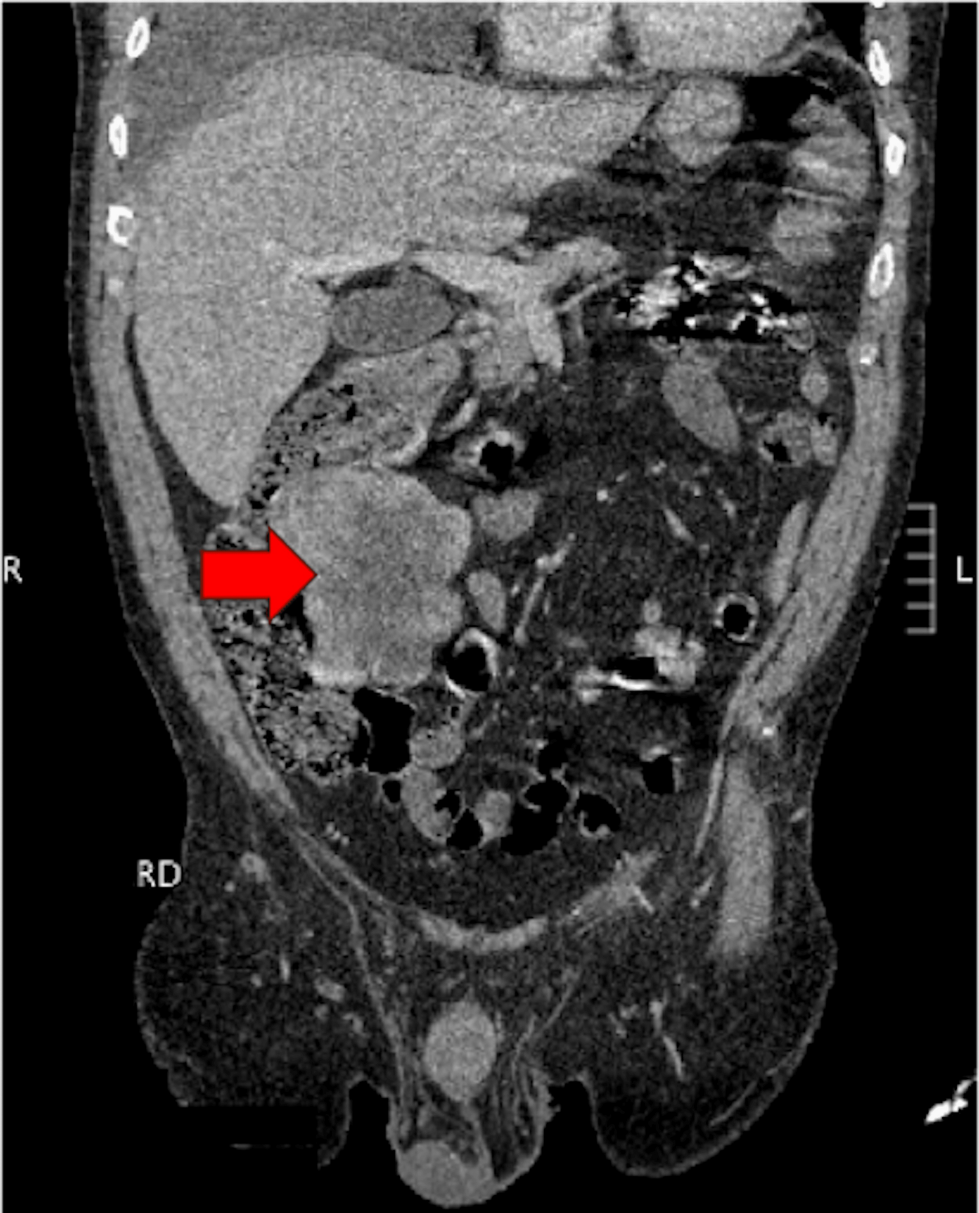
Coronal view of contrast-enhanced computed tomography (CT) of the abdomen demonstrating a well-circumscribed mesenteric mass adjacent to the ascending colon (red arrow). The image highlights the size and location of the lesion, consistent with metastatic involvement from an inflammatory myofibroblastic tumor.

Further staging with CT of the abdomen and pelvis demonstrated a right mid-abdominal mesenteric mass along the ascending colon with satellite nodules, as well as a heterogeneous soft tissue mass posterior to the right hip joint measuring 4 × 6.1 cm and a right perineal mass measuring 4.2 × 3 cm, findings concerning for metastatic disease. The mesenteric mass is shown in Figure 2.

The pulmonary embolism was treated with intravenous anticoagulation. The patient subsequently underwent endobronchial ultrasound (EBUS) with biopsy of the right endobronchial mass. He also underwent fiberoptic bronchoscopy with therapeutic mucus aspiration and cryoablation with multiple rounds of tumor debulking. Bronchoscopy revealed extensive necrotic mucosal changes throughout the right-sided airways. 

**Figure 3:**
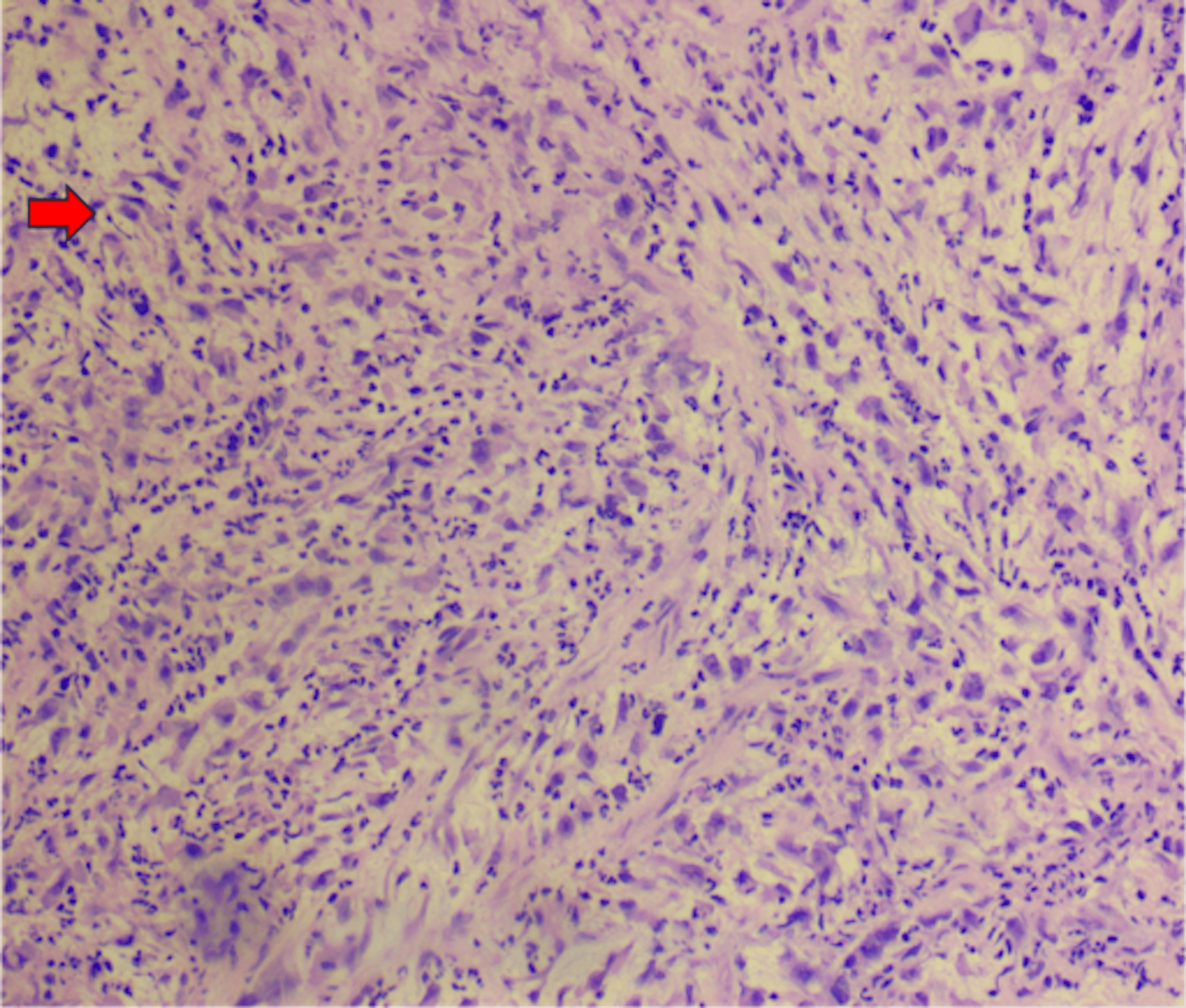
Hematoxylin and eosin (H&E)-stained section of the right mainstem bronchus biopsy demonstrating a malignant spindle cell neoplasm with sarcomatoid and myxoid features. Magnification, 100×. The spindle-shaped tumor cells with a myxoid background are highlighted, consistent with a high-grade sarcomatoid process. The red arrow indicates tumor cell clusters highlighted by the H&E staining pattern.

**Figure 4:**
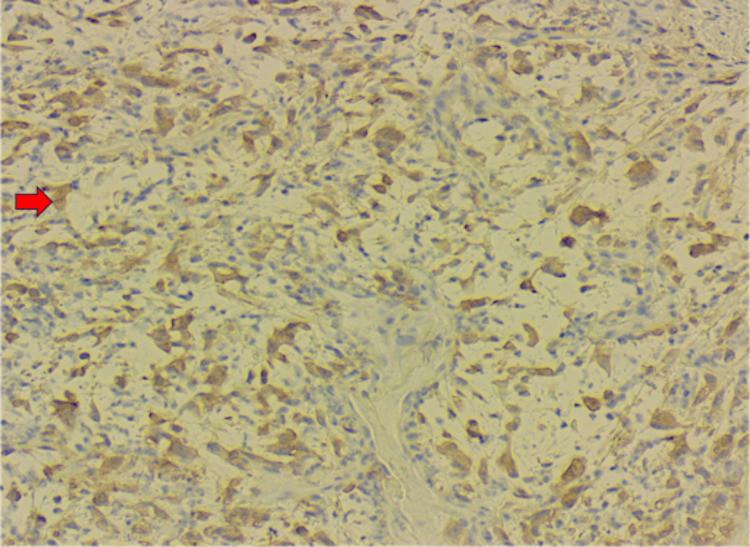
Immunohistochemical staining of the right mainstem bronchus biopsy demonstrating strong cytoplasmic positivity for ALK-1 in tumor cells, supporting the diagnosis of inflammatory myofibroblastic tumor. Magnification, 100×. Red arrow highlights tumor cell expression of ALK-1, consistent with underlying ALK gene rearrangement and supportive of a neoplastic process.

Histopathologic evaluation of the bronchoscopic biopsy revealed a malignant spindle cell neoplasm with sarcomatoid and myxoid features (Figure 3). Immunohistochemical analysis demonstrated positivity for *ALK-1* and CD68 and negativity for AE1/3, MOC31, p40, TTF-1, SMA, SOX10, calretinin, S100, and desmin, supporting the diagnosis of IMT. Representative immunohistochemical staining is shown in Figure 4.

**Figure 5:**
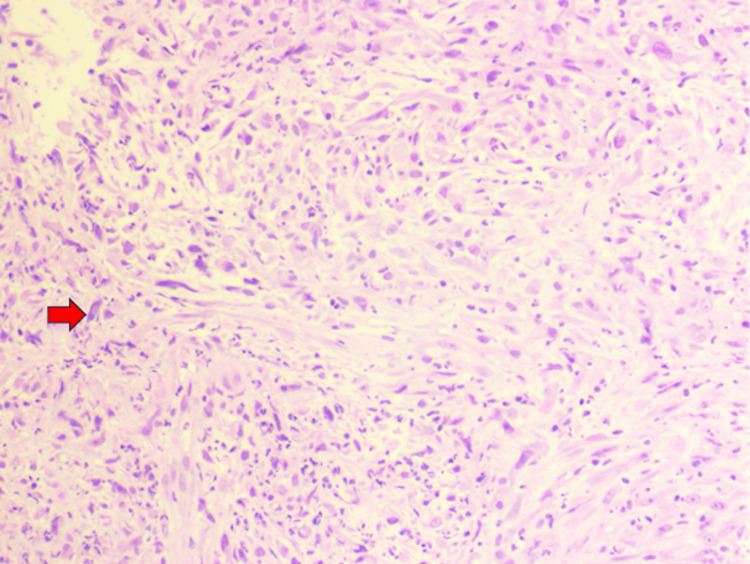
Hematoxylin and eosin (H&E)-stained section of the ascending colon biopsy demonstrating a malignant spindle cell neoplasm with sarcomatoid features, consistent with metastatic inflammatory myofibroblastic tumor. Magnification, 100×. The red arrow highlights infiltrating spindle tumor cells within the colonic tissue.

Given the imaging findings of a mesenteric mass, the patient underwent colonoscopy, which revealed a fungating, non-obstructing mass in the ascending colon measuring approximately 4 cm and involving one-third of the luminal circumference. Biopsy of this lesion demonstrated a malignant neoplasm with sarcomatoid features, as shown in Figure 5, with immunohistochemistry positive for *ALK-1*, CD68, and vimentin and negative for CK7, CK20, CDX2, CD117, and SATB2. These findings were consistent with the bronchial lesion and supported the diagnosis of IMT with extensive metastatic disease.

In summary, the combination of a rapidly progressive obstructing endobronchial mass, multifocal systemic lesions, and a spindle cell neoplasm with *ALK* positivity on immunohistochemistry were key diagnostic features supporting metastatic IMT.

The patient was planned for repeat EBUS with bronchial stent placement and additional tissue sampling for oncologic treatment planning. However, his hospital course was complicated by progressive respiratory distress requiring transfer to the intensive care unit (ICU). Repeat chest imaging demonstrated complete right lung opacification, significantly worsened from prior studies.

During his ICU course, the patient required endotracheal intubation, placement of a right mainstem bronchial stent, and two right-sided chest tubes without significant clinical improvement. His hospitalization was further complicated by pneumonia and gastrointestinal bleeding. Given his rapidly progressive disease and poor overall prognosis, the patient and his family elected to pursue comfort-focused care. He subsequently died two months after his initial presentation.

## Discussion

IMT is a rare mesenchymal neoplasm that typically affects children and young adults, most commonly arising in the lung, abdomen, or pelvis. IMT accounts for less than 1% of all soft tissue tumors [3]. Adult presentations are uncommon, and metastatic disease is exceedingly rare. 

Our case highlights an aggressive, widely metastatic IMT in a 56-year-old male, presenting as an obstructing right upper lobe bronchial mass with multiorgan involvement, including the colon, perineum, and soft tissue near the hip. To our knowledge, such extensive metastatic involvement in an adult with pulmonary IMT has been rarely reported. Fewer than 50 cases of metastatic IMT have been reported in the English literature, and metastatic disease arising from pulmonary IMT in adults is particularly rare, with only isolated case reports described [4]. Metastatic spread to the colon mucosa and perineal soft tissues is especially unusual.

Another notable aspect of this case is the uncertainty regarding the primary site of disease. Both pulmonary and intra-abdominal masses were identified at presentation, and the patient was found to have involvement of the bronchial tree as well as a fungating lesion within the ascending colon. As a result, it is difficult to definitively determine whether the tumor originated in the lung with subsequent gastrointestinal metastasis or whether the colonic lesion represented the primary site with pulmonary involvement. However, several features favor a pulmonary primary tumor. The patient presented with a large obstructing right hilar mass with extensive endobronchial involvement and mediastinal lymphadenopathy, suggesting a primary pulmonary process. Additionally, the lung is one of the most frequently reported primary sites of IMT, whereas primary gastrointestinal IMT is comparatively less common [5]. Given the extensive intrathoracic disease burden and the higher reported incidence of pulmonary IMT, the clinical presentation in this case most likely represents a primary pulmonary IMT with widespread metastatic dissemination.

Diagnosis of IMT can be challenging because of overlapping histopathologic features with other spindle cell neoplasms, including sarcomatoid carcinoma, leiomyosarcoma, and malignant peripheral nerve sheath tumor [6]. In this case, the initial clinical and radiographic presentation raised a strong suspicion for primary lung carcinoma or aggressive lymphoma. However, the absence of epithelial marker expression (AE1/3, MOC31, p40, and TTF-1) argued against sarcomatoid carcinoma, while negative desmin and SMA made leiomyosarcoma less likely. Similarly, a lack of S100 and SOX10 expression reduced the likelihood of a malignant peripheral nerve sheath tumor. In contrast, strong *ALK-1* positivity supported the diagnosis of IMT, as *ALK* rearrangements are identified in a substantial subset of cases and are uncommon in most histologic mimics.

Pulmonary IMTs account for approximately 1% of all lung tumors [6]. Therefore, immunohistochemical analysis plays a crucial role in establishing the diagnosis. In our case, positivity for *ALK-1*, CD68, and vimentin, along with the absence of epithelial, smooth muscle, neural, and melanocytic markers, strongly supported the diagnosis of IMT. Although molecular confirmation with fluorescence in situ hybridization or next-generation sequencing was not performed, these techniques can further validate ALK rearrangements and may help identify additional actionable gene fusions in diagnostically challenging cases.

Adult IMTs with rapid progression and obstructing endobronchial lesions may clinically mimic primary lung malignancies, emphasizing the need for early biopsy and comprehensive immunohistochemical evaluation. Additionally, occupational exposures, such as this patient’s prolonged construction work with significant dust and cement exposure, may further complicate the clinical presentation, although a causal relationship with IMT has not been established.

The clinical course in this case was notable for rapid progression over a short time interval, with worsening airway obstruction requiring repeated bronchoscopic interventions and eventual complete right lung opacification on follow-up imaging. Despite endobronchial tumor debulking and stent placement, the patient experienced progressive respiratory failure, suggesting limited therapeutic benefit from local control measures in the setting of extensive disease burden. While pulmonary emboli were present at diagnosis and may have contributed to overall clinical decline, the patient’s deterioration was primarily driven by aggressive tumor progression and respiratory compromise.

The primary treatment for IMT is complete surgical resection when feasible, particularly in localized disease [1]. For advanced, recurrent, or metastatic disease, targeted therapy has emerged as an important treatment modality. The National Comprehensive Cancer Network (NCCN) recommends *ALK* inhibitors as first-line systemic therapy for patients with unresectable or metastatic IMT harboring *ALK* rearrangements [7,8]. In this case, despite *ALK* positivity, targeted therapy was not initiated due to the patient’s rapid clinical deterioration and poor functional status, which precluded systemic treatment.

IMT should be considered in the differential of adult spindle cell tumors, even in cases with extensive metastatic disease. Prompt recognition and accurate diagnosis are essential, as the clinical course may be aggressive and the therapeutic approach differs significantly from other malignancies. Although IMTs are typically indolent and occur in younger populations, this case demonstrates that they can rarely present as a rapidly progressive and widely metastatic disease in older adults.

## Conclusions

This case illustrates a rare and aggressive presentation of IMT in an older adult, characterized by extensive pulmonary involvement and widespread metastases. It underscores the significant diagnostic challenges posed by sarcomatoid morphology and the importance of comprehensive immunohistochemical evaluation, particularly *ALK* testing, in distinguishing IMT from other spindle cell malignancies.

While IMT is typically considered a tumor of intermediate biologic potential, this case highlights its capacity for rapid progression and poor outcomes in atypical presentations. Early recognition is critical, as prompt and accurate diagnosis not only informs prognosis but may also expand therapeutic options. In particular, identification of *ALK* rearrangements has important clinical implications, as patients may potentially benefit from targeted therapies such as *ALK* inhibitors, as reported in other studies.
